# Transcranial Photobiomodulation Attenuates Neurogenic Inflammation and Sensory Hypersensitivity in a Nitroglycerin‐Induced Migraine Model: Toward a Nonpharmacological Therapy

**DOI:** 10.1155/prm/1530362

**Published:** 2026-10-17

**Authors:** Sareh Kazmi, Seyed Mahdi Vatandoust, Seyed Zanyar Athari, Asal Ebrahimian, Afshin Moradi, Javad Mahmoudi, Saeed Sadigh-Eteghad, Mojgan Rajabi, Sina Akhbari-Shojaei, Ramtin Neshan-Alipour, Abdollah Nozad, Moein Hosseinkhani, Leila Hosseini, Michael R. Hamblin, Fereshteh Farajdokht

**Affiliations:** ^1^ Neurosciences Research Center Tabriz University of Medical Sciences Tabriz Iran tbzmed.ac.ir; ^2^ Student Research Committee Tabriz University of Medical Sciences Tabriz Iran tbzmed.ac.ir; ^3^ Drug Applied Research Center Tabriz University of Medical Sciences Tabriz Iran tbzmed.ac.ir; ^4^ Research Center of Psychiatry and Behavioral Sciences Tabriz University of Medical Sciences Tabriz Iran tbzmed.ac.ir; ^5^ Laser Research Centre University of Johannesburg Doornfontein South Africa uj.ac.za

**Keywords:** CGRP, migraine, neuroinflammation, neuropeptides, nitroglycerin, photobiomodulation

## Abstract

**Background:**

Migraine is a debilitating neurological condition, characterized by headache, photophobia, and heightened mechanical/thermal sensitivity. Current pharmacological therapies often lead to incomplete relief and adverse effects, necessitating the use of noninvasive alternatives. This study evaluated transcranial photobiomodulation (PBM) in a nitroglycerin (NTG)‐induced migraine model in male mice.

**Methods:**

Mice were assigned to Sham, NTG, NTG + Sumatriptan (SUMA; 600 µg/kg, s.c.), NTG + PBM, and NTG + PBM + SUMA groups. Over 9 days, NTG (5 mg/kg) was injected intraperitoneally every other day to develop an animal model of chronic migraine. The PBM protocol involved daily irradiation (810 nm) on the occipital bone of the head for 5 s over 9 days. The animals underwent behavioral testing for photophobia, mechanical allodynia, and thermal sensitivity. The trigeminal nucleus caudalis (TNC) was assessed for miR‐155 expression by real‐time PCR and protein expression of phospho‐NF‐κB, IL‐1*β*, transient receptor potential ankyrin 1 (TRPA1) and vanilloid 1 (TRPV1), calcitonin gene–related peptide (CGRP), c‐Fos, and pituitary adenylate cyclase–activating polypeptide (PACAP) using immunoblotting.

**Results:**

Behavioral assessment revealed that PBM significantly reduced head scratching and improved exploratory activity, decreased light aversion, reversed periorbital/hindpaw mechanical allodynia, and prolonged thermal withdrawal latency compared to the NTG group. Moreover, PBM downregulated miR‐155 expression and decreased levels of inflammatory mediators and migraine‐associated neuropeptides in the TNC. Notably, combined PBM and SUMA treatment resulted in improvements in behavioral and molecular outcomes compared to either treatment alone.

**Conclusion:**

These results indicate that PBM inhibited migraine‐like behaviors and decreased the expression of numerous molecular markers that are linked to neuroinflammation and trigeminal activation in the TNC. These findings establish a foundation for additional research on transcranial PBM as a noninvasive method for modulating migraine‐related behavioral responses.

## 1. Introduction

Migraine is a widespread and incapacitating disorder distinguished by intermittent, severe cephalic pain, often concomitant with symptoms such as nausea, fatigue, and sensory hypersensitivity [1], affecting nearly 14%–15% of the global population [2, 3]. Treatment of this disorder is challenging because of its complex and multifactorial biology, recurring pattern, and the complex brain networks implicated in its pathophysiology [4].

Trigeminal ganglion axons carry sensory input from the meninges and intracranial blood vessels to the trigeminal nucleus caudalis (TNC) and the upper cervical spinal cord for processing. Second‐order nociceptive neurons provide signals to the thalamus, where third‐order neurons send signals to migraine‐related cortical areas [5, 6]. Trigeminal nociceptors express transient receptor potential (TRP) channels, predominantly ankyrin 1 (TRPA1) and vanilloid 1 (TRPV1), which upon activation induce the release of various pro‐inflammatory agents and vasodilatory neuropeptides, including calcitonin gene–related peptide (CGRP), pituitary adenylate cyclase activating polypeptide (PACAP), and substance P from sensory nerve endings [7, 8]. This process increases nitric oxide (NO) synthesis and neurogenic inflammation, which vasodilates meningeal blood vessels and produces peripheral and central pain hypersensitivity [9–11].

Nitroglycerin (NTG), a NO donor, reliably triggers migraine‐like attacks in both humans and rodents, reflected by mechanical allodynia, photophobia, and neuroinflammation [12–15]. Repeated NTG exposure upregulates microRNA‐155 (miR‐155) in the TNC, promoting microglial polarization toward a pro‐inflammatory phenotype via NF‐κB, ultimately leading to pain hypersensitivity [16, 17]. In contrast, blocking miR‐155 in animal pain models reverses the rise in pro‐inflammatory mediators and the decline in tactile and thermal pain hypersensitivity [17, 18].

Although migraine is prevalent and can be severe, it is frequently overlooked and poorly managed. Current treatments, mainly nonsteroidal anti‐inflammatory drugs and triptans, often provide insufficient relief and can cause side effects [19–21]. Moreover, high costs limit access to newer therapies, such as CGRP antagonists [22]. Although sumatriptan (SUMA) alleviates migraine in many patients, its 20%–40% failure rate, cardiovascular contraindications, and risk of medication‐overuse headache [23] highlight the necessity for safer and more effective alternative therapies.

Photobiomodulation (PBM) is a new noninvasive approach that modulates cell biochemistry using red or near‐infrared light [24, 25]. PBM has shown promise in treating orofacial pain [26] and neuropathic pain conditions like trigeminal neuralgia [27]. The analgesic effect of PBM is mediated by temporarily blocking nerve conduction (Aδ and C fibers), improving blood flow, decreasing pain mediators (bradykinin), and increasing endogenous opioid synthesis (beta‐endorphin) [28, 29]. In particular, PBM shows potential in modulating neuroinflammation, an important element of migraine pathogenesis, by reducing levels of TNF‐*α*, IL‐1*β*, and IL‐6 [30, 31]. Moreover, application of 904 nm PBM in a neuropathic pain model led to a decrease in the levels of substance P and TRPV1 proteins in the dorsal root ganglia [32].

In light of these findings, PBM may represent a promising nonpharmacological approach that could complement conventional pharmacotherapy for migraine management. Moreover, combining PBM with triptans may produce complementary therapeutic effects by activating distinct mechanisms that are involved in the pathophysiology of migraines. Thus, the present study aimed to evaluate the effects of transcranial PBM on pain sensitivity, light aversion, and neuroinflammatory signaling in the TNC in an NTG‐induced migraine model. Furthermore, we also examined whether the combination of PBM and SUMA enhances therapeutic efficacy compared to either intervention alone.

## 2. Materials and Methods

### 2.1. Ethics Approval

The regulations established by Tabriz University of Medical Sciences regarding the treatment of animals were strictly followed throughout the entire procedure. Animal procedures were conducted in accordance with the relevant institutional and national regulations regarding the care and use of laboratory animals and received approval from the Ethics Committee of Animal Research at Tabriz University of Medical Sciences (Approval number: IR.TBZMED.VCR.REC.1400.247). All animal procedures were performed in compliance with the ARRIVE guidelines.

### 2.2. Animals and Experimental Groups

Sixty adult male C57BL/6 mice (25–30 g) that had never been used for experiments before were acquired from the experimental animal center of Tabriz University of Medical Sciences. The mice were maintained at a temperature of 23 ± 2°C, humidity of 50 ± 5%, and had unrestricted access to standard laboratory chow and water *ad libitum*, with a 12‐h light–dark pattern. Before beginning the procedures, mice were acclimated for a duration of 1 week. Exclusion criteria included the presence of disease, aberrant baseline nociceptive thresholds (> 2 SD from the group mean), significant adverse responses to NTG, or reaching humane endpoints (such as > 20% weight loss, immobility, or self‐injury). Next, healthy animals with normal activity, appearance, and baseline behavioral responses were randomly allocated to five separate research groups, with 8–12 animals in each group, as follows: Sham, NTG, NTG + SUMA, NTG + PBM, and NTG + PBM + SUMA. The sample size was established based on previous research utilizing similar NTG‐induced migraine models to ensure sufficient statistical power. All animals successfully completed the study, and no data were excluded from the analysis. Figure 1 illustrates the study protocol. All behavioral testing, tissue processing, and data analysis were conducted by investigators blinded to group allocation. To reduce possible confounders, all procedures were carried out at the same time of day in the same environment, and cage locations were changed regularly to prevent location effects.

**FIGURE 1:**
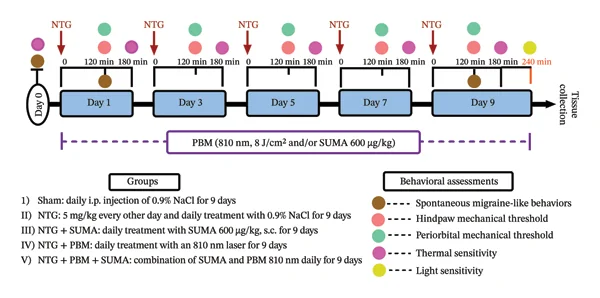
Study design. NTG, nitroglycerin; SUMA, sumatriptan; PBM, photobiomodulation.

### 2.3. Migraine Induction and Treatments

The NTG stock solution (Caspian Tamin Pharmaceutical Company, Iran), with a concentration of 5 mg/mL, was prepared in a mixture of 30% alcohol, 30% propylene glycol, and distilled water. NTG stock solution was diluted in saline (0.9% NaCl) immediately before use and injected intraperitoneally (i.p.) at a dosage of 5 mg/kg every other day [33]. As there were no noticeable differences in mechanical sensitivity between mice administered 0.9% saline and the NTG solvent in the pilot study, the vehicle used for injection in the sham group was 0.9% saline (data are not shown). The mice were administered five injections of NTG or vehicle on Days 1, 3, 5, 7, and 9. Each injection had a maximum volume of 200 µL.

The mice in both the sham and NTG groups were given daily i.p. injections of 0.9% NaCl for a total of 9 days. The NTG + SUMA group was given daily i.p. injections of SUMA, using an injectable form of SUMA succinate (Caspian Tamin Pharmaceutical Company, Iran) at a dosage of 600 µg/kg [13] for 9 days. The mice in the NTG + PBM group were subjected to daily treatment with an 810 nm laser for 9 days. Moreover, the NTG + PBM + SUMA group received a combination of SUMA and PBM 810 nm daily for 9 days.

### 2.4. PBM

The PBM groups were exposed to a near‐infrared diode laser (Thor Photomedicine, Chesham, UK) at a wavelength of 810 nm, a maximum optical power output of 200 mW, a beam area of 0.0364 cm^2^, a duty cycle 88% (pulse width 88 ms), an average irradiance 4.75 W/cm^2^, and a pulse frequency of 10 Hz on a daily schedule for 9 days to provide a sustained PBM effect. To optimize light penetration to the target tissue and reduce light scattering, the hair in the application area was shaved, and the total treatment time was 5 s per session, corresponding to a scalp surface fluence of 23.75 J/cm^2^. Based on previous reports of tissue penetration for 810 nm light in mice, the energy density delivered to the cortical surface after attenuation through the scalp and skull was estimated to be approximately 8 J/cm^2^ [34]. PBM sessions were performed on awake, gently restrained mice. The laser probe was positioned perpendicularly posterior to lambda on the occipital bone at approximate stereotaxic coordinates (AP–7.5 mm from bregma) to target the TNC, which is a key region involved in migraine pathophysiology [35]. In the Sham and NTG groups, animals underwent identical handling, restraint, and probe placement procedures following the same schedule; however, the laser device remained switched off, and no light was delivered (sham irradiation). There are currently no established parameters for PBM therapy in a migraine model in rodents. This wavelength was chosen based on prior studies showing that transcranial application of an 810 nm laser significantly increased pain thresholds, with peak effects at 2–3 h and persisting for up to 24 h [36, 37]. In addition, energy densities within the therapeutic window for PBM are widely acknowledged to be within the range of 1–10 J/cm^2^. In order to reduce thermal burden and optimize neuromodulatory effects, pulsed irradiation at 10 Hz was employed, as this frequency has been demonstrated to increase tissue repair and reduce inflammation more effectively than continuous or higher frequency modes [24, 38].

### 2.5. Behavioral Tests

All behavioral experiments were performed by an investigator who was unaware of group allocation between 9:00 a.m. and 12:00 p.m. to minimize variability related to circadian rhythms.

#### 2.5.1. Spontaneous Pain–Related Behaviors

Each mouse was separately placed in a 30 × 40 cm transparent Plexiglas box for 10 min before (Day 0, baseline) and 120 min after the first (Day 1) and fifth injections (Day 9) of NTG. To ensure a consistent and mild illumination, the ambient light levels were adjusted to approximately 100 lux. The apparatus was meticulously cleaned with 70% ethanol between trials to eliminate any odor indicators. The experimenter remained outside the testing chamber for each session in order to reduce the potential disruption to the animals. A video recorder was set up in front of the device, and for 10 minutes, the behaviors of the animal were documented and then analyzed using EthoVision software. The subsequent parameters were recorded: the number of head scratches and grooming time (on the face or body), which were assessed as complementary measures of spontaneous pain‐related behavior, and the distance traveled and time spent in exploratory activities (cage climbing and rearing), which were used to evaluate general locomotor and exploratory activity.

#### 2.5.2. Hindpaw and Periorbital Mechanical Sensitivity

On Days 1, 3, 5, 7, and 9 before injection (basal reactions) and 120 min after injection of NTG or vehicle (posttreatment responses), mechanical sensitivity was evaluated using Von Frey filaments. All animals were given 30 min to acclimate to the testing chamber before beginning the procedure. Every behavioral test was carried out in a quiet room by an investigator unaware of the research groups, between 9 and 11 a.m.

Before testing the hindpaw mechanical sensitivity threshold, each mouse was placed in a cage with a mesh floor. A set of von Frey monofilaments (bending force comprising 0.008 g to 2 g) was perpendicularly inserted through the mesh floor to contact the plantar surface of the mouse hindpaw. Paw movements such as lifting, shaking, or licking in response to stimulation were considered positive responses to the stimulus. The test was continued with a thinner filament if a positive response was observed, and a thicker filament was used if no reaction was detected. The filament was applied to each paw three times, with a minimum interval of 1 min between each test. The hindpaw mechanical threshold was established as the least amount of force needed to produce a minimum of two positive responses from three tests [39].

The periorbital sensitivity threshold was measured by placing the mouse in a glass container that allowed the head to be exposed. After acclimation in the restraining apparatus for 5 min, von Frey monofilaments were applied to the center and upper portions of the eye orbit bilaterally. A positive reaction was characterized by rapid head retraction or shaking or facial rubbing with the same‐side forelimb [39].

#### 2.5.3. Thermal Sensitivity

To assess the thermal sensitivity of the paws, the mice were placed on a hot plate at a temperature of 55°C on Day 0 (baseline response) and on Days 1, 3, 5, 7, and 9 of the experiment 180 min after NTG injection. The time before the mouse withdrew the limbs or jumped off the plate was recorded. A 30‐s time limit was established to avoid any tissue injury [40].

#### 2.5.4. Sensitivity to Light

In order to assess photophobia or sensitivity to light, a light/dark box was employed on Day 9, 4 hours following the final NTG injection. The light/dark box (50 × 40 × 30 cm) consisted of two chambers. One chamber was transparent, without a door, and illuminated by a 1000 lux light that served as the light chamber. The dark box was entirely covered in black paint and was connected to the light chamber by a small 12 × 12 cm opening. The animal was individually positioned in the middle of the light chamber on the testing day. The connecting door was left open, allowing the animal to access the entire apparatus, and the behavior of the mouse was tracked for 10 min. The proportion of time spent in the light chamber and the latency in entering the dark chamber in the initial attempt were recorded [41].

### 2.6. Isolation of TNC

At the final step of the experiment, mice were anesthetized via intraperitoneal administration of pentobarbital (200 mg/kg), and 10 mice from each group were euthanized. The TNC tissue was isolated from the medulla oblongata, promptly cryopreserved in liquid nitrogen, and subsequently maintained at −70°C for molecular evaluation.

### 2.7. Quantitative Real‐Time PCR

The TNC tissues were subjected to total RNA extraction by applying Trizol reagent (Roche). The spectrophotometric examination of the RNA concentration and quality was carried out using a NanoDrop spectrophotometer (Thermo Fisher Scientific, USA). Next, the TaqMan miRNA Reverse Transcription Kit (Takara, Tokyo, Japan) was used to reverse transcribe miR‐155‐5p. Using the LightCycler 96 Instrument (Roche, Germany), real‐time polymerase chain reaction assays were carried out with SYBR Green PCR Master Mix (Takara, Tokyo, Japan). For miRNA 155, the primer sequences were: forward: 5′‐CGGCGGTTAATGCTAATTGTGAT‐3′ and reverse: 5′‐GTGCAGGGTCCGAGGT‐3′, and for U6 were forward 5′‐TGCGGGTGCTCGCTTCGGCAGC‐3′ and reverse: 5′‐CCAGTGCAGGGTCCGAGGT‐3′. The corresponding values of miR‐155 expression were estimated using the 2−ΔΔCt method, normalized by the U6 reference gene, and compared to the control sample. The total volume of each reaction was 10 µL and was conducted in triplicate.

### 2.8. Immunoblotting

The TNC tissue samples were homogenized using an ultrasonic homogenizer in cold lysis buffer Pro‐PRETM (iNtRON Biotechnology, Korea) comprising a protease inhibitor cocktail. The resulting mixture was then centrifuged at 13000 rpm (Hettich Universal 320R, Germany) for 15 min at 4°C. The protein contents in the collected supernatant were measured spectrophotometrically using the BCA method (iNtRON Biotechnology, Korea). Following this, proteins were separated by SDS‐PAGE electrophoresis and subsequently transferred to a PVDF membrane. After the membranes were blocked with 5% milk, they were treated with the primary antibodies listed below: anti‐PACAP (Cat No.: ab181205, Abcam), anti‐c‐Fos (Cat No.: ab222699, Abcam), anti‐TRPA1 (Cat No.: NB110‐40763SS, Novus Biologicals), anti‐TRPV1 (Cat No.: 22686‐1‐AP, PTGLAB), anti‐CGRP (Cat No.: 14959T, Cell Signal), anti‐IL‐1*β* (Cat No.: ab254360, Abcam), anti‐NF‐κB p65 phospho S536 (Cat No.: ab76302, Abcam), and anti‐*β*‐actin (Cat No: ab8227, Abcam). The incubation was done overnight at 4°C. Next, the membranes were washed three times and exposed to goat anti‐rabbit IgG H&L (HRP) (Cat No: ab6721; Abcam) secondary antibody for 1 h at ambient temperature. The bands were detected using an enhanced chemiluminescence kit, and the quantification was done by densitometric analysis using the gel analyzer Version 2010a software (NIH, USA). Original, uncropped Western blot images are provided in Supporting Figure S1.

### 2.9. Statistical Analysis

Data are presented as mean ± SEM in this study. Graphs and statistics were performed with GraphPad Prism 9 (GraphPad Software Inc., San Diego, California, USA). The Kolmogorov–Smirnov test was used to test the normality of data before statistical analysis. Behavioral outcomes measured over time were analyzed using a two‐way repeated‐measures ANOVA with treatment as the between‐subjects factor and time as the within‐subjects factor, followed by Tukey’s multiple‐comparisons test with adjusted *p* values. One‐way ANOVA with Tukey’s multiple comparisons test was used for single time point outcomes. Effect‐size estimates (partial eta squared, ηp2) and 95% confidence intervals for pairwise mean differences are reported in Supporting Tables S1–S3. The *p* value was determined to be less than 0.05 in order to establish statistical significance.

## 3. Results

### 3.1. PBM Alone or in Combination With SUMA Attenuated NTG‐Associated Spontaneous Pain‐Related Behavioral Responses

To examine the immediate and long‐lasting effects of NTG and the treatments on spontaneous pain‐related behavioral responses, we measured locomotor activity (distance traveled), exploratory activity, head scratching, and grooming behavior before NTG or vehicle administration and 2 h after the first and fifth injections (Days 1 and 9). The results showed no significant differences in these measurements before the injections (baseline responses).

However, the posttreatment analysis revealed that the NTG group exhibited a decrease in travel distance (Figure 2a, *p* < 0.001 on both days) and exploration time (Figure 2b, *p* < 0.001 on both days), as well as an increase in head scratching (Figure 2c, *p* < 0.01 on Day 1 and *p* < 0.001 on Day 9) and grooming time (Figure 2d, *p* < 0.001 for both days) in comparison to the sham group. On Day 1, combined PBM + SUMA treatment increased distance traveled and exploration time (*p* < 0.05 for both outcomes) and reduced head scratching (*p* < 0.001) and grooming time (*p* < 0.05) compared with the NTG group. PBM and SUMA monotherapies also reduced head scratching compared with NTG (*p* < 0.05 for both treatments). Furthermore, PBM and SUMA, administered alone or in combination, increased distance traveled (*p* < 0.05 for monotherapies and *p* < 0.001 for the combination) and exploration time (*p* < 0.05 for monotherapies and *p* < 0.01 for the combination), while reducing head scratching (*p* < 0.001 for all treatments) and grooming time (*p* < 0.05 for monotherapies and *p* < 0.01 for the combination). These findings indicate that PBM and SUMA, administered either individually or concurrently, attenuated NTG‐associated changes in spontaneous pain‐related behavioral responses.

**FIGURE 2:**
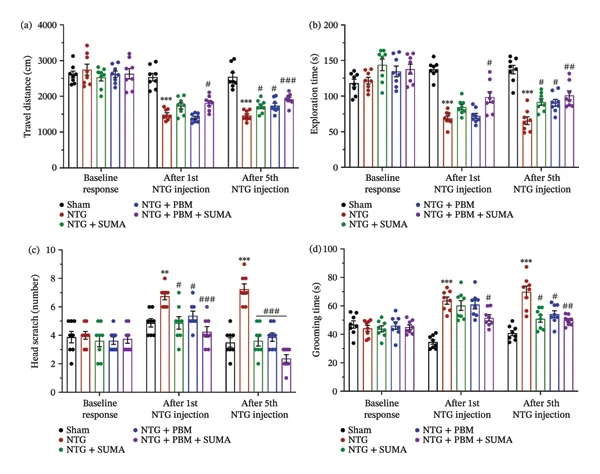
Effects of PBM or SUMA, alone or combined, on (a) travel distance, (b) exploration time, (c) head scratch number, and (d) grooming time before and 120 min after the first and the fifth NTG injection in the experimental groups. The results are shown as mean ± SEM for eight animals for each group at different times. ^∗∗^ *p* < 0.01 and ^∗∗∗^ *p* < 0.001 compared to the Sham group. ^#^ *p* < 0.05, ^##^ *p* < 0.01, and ^###^ *p* < 0.001 compared to the NTG group. NTG, nitroglycerin; SUMA, sumatriptan; PBM, photobiomodulation.

### 3.2. PBM Alone or in Combination With SUMA Attenuated NTG‐Evoked Mechanical Hyperalgesia

Two‐way repeated‐measures ANOVA of baseline hindpaw and periorbital mechanical sensitivity thresholds revealed significant effects of treatment [hindpaw: *F*_(4,  45)_  = 73.37, *p* < 0.001; periorbital: *F*_(4,  45)_ = 31.93, *p* < 0.001], day [hindpaw: *F*_(3.561,  160.2)_ = 22.08, *p* < 0.001; periorbital threshold: *F*_(3.955,  178.0)_ = 17.86, *p* < 0.001], and interaction of treatment and day [hindpaw: *F*_(16,  180)_ = 2.846, *p* = 0.004; periorbital: *F*_(16,  180)_ = 2.415, *p* = 0.0026]. Moreover, two‐way repeated‐measures ANOVA of posttreatment responses of hindpaw and periorbital mechanical sensitivity thresholds indicated significant effects of treatment [hindpaw threshold: *F*_(4,  45)_ = 61.92, *p* < 0.001; periorbital threshold: *F*_(4,  45)_ = 52.19, *p* < 0.00 1], a significant effect of day for hindpaw threshold (*F*_(3.235,  145.6)_ = 4.004, *p* = 0.007) but no main effect of day for periorbital threshold (*F*_(3.406,  153.3)_ = 1.195, *p* = 0.3150), and no significant treatment × day interaction [hindpaw threshold: *F*_(16, 180)_ = 0.3276, *p* = 0.9936; periorbital threshold: *F*_(16,  180)_ = 0.2094, *p* = 0.9996)].

NTG‐injected mice exhibited a marked reduction in baseline hindpaw mechanical thresholds compared to the sham animals on Days 3, 5, 7, and 9 (Figure 3a; *p* < 0.001). However, treatment with PBM (*p* < 0.05 on Days 3–5; *p* < 0.01 on Days 7–9), SUMA (*p* < 0.05 on Days 3–5; *p* < 0.01 on Day 7; *p* < 0.001 on Day 9), or their combination (*p* < 0.05 on day 1; *p* < 0.01 on Days 5 and 9; *p* < 0.001 on Day 7) significantly increased hindpaw thresholds, indicating a partial reversal of mechanical hypersensitivity.

**FIGURE 3:**
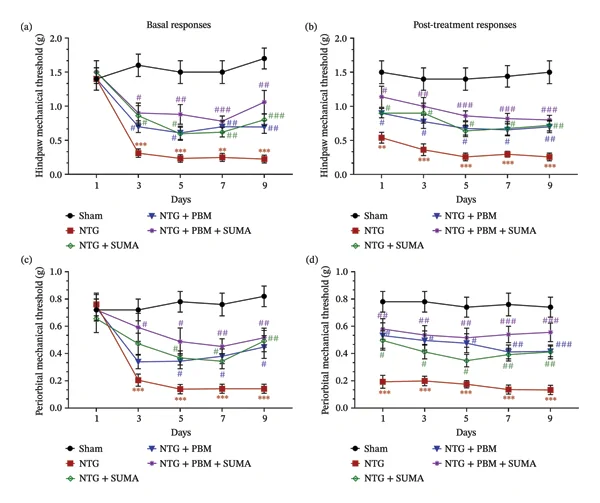
Treatment with PBM and SUMA, alone or combined, improved NTG‐induced mechanical hyperalgesia. Basal (left panel) and posttreatment (right panel) mechanical pain threshold of the hindpaw. (a) Basal responses. (b) Posttreatment responses and periorbital area. (c) Basal responses. (d). Posttreatment responses in NTG‐treated mice. The results are shown as mean ± SEM for 10 animals for each group at different times. ^∗∗^ *p* < 0.01 and ^∗∗∗^ *p* < 0.001 compared to the Sham group. ^#^ *p* < 0.05, ^##^ *p* < 0.01, and ^###^ *p* < 0.001 compared to the NTG group. NTG, nitroglycerin; SUMA, sumatriptan; PBM, photobiomodulation.

Posttreatment assessments (120 min after NTG injection or 90 min after therapy) confirmed that repeated NTG administration produced robust and sustained mechanical allodynia in the hindpaw (Figure 3b; *p* < 0.01 on Day 1 and *p* < 0.001 on Days 3–9). Administration of SUMA or PBM alone significantly elevated hindpaw thresholds (*p* < 0.05 on Days 1–7; *p* < 0.01 on Day 9), whereas combined PBM + SUMA treatment increased thresholds on Days 1–5 (*p* < 0.01) and Days 7–9 (*p* < 0.001).

A similar pattern was observed for periorbital mechanical thresholds. NTG‐injected mice showed a significant baseline decrease relative to the sham controls on Days 3, 5, 7, and 9 (Figure 3c; *p* < 0.001). PBM treatment (*p* < 0.05 on Days 5–9), SUMA (*p* < 0.05 on Days 5 and 7; *p* < 0.01 on Day 9), and combined therapy (*p* < 0.05 on Days 1 and 3; *p* < 0.01 on Days 7 and 9) significantly elevated periorbital thresholds during baseline testing.

Posttreatment analysis revealed that NTG administration produced a consistent and pronounced decrease in periorbital thresholds across all test days (Figure 3d; *p* < 0.001). Treatment with SUMA or PBM alone significantly attenuated this effect (*p* < 0.05 on Days 1–5 and *p* < 0.0 1 on Day 7 for both monotherapies; *p* < 0.01 on Day 9 for SUMA and *p* < 0.001 on Day 9 for PBM). Combined PBM + SUMA treatment also significantly increased periorbital thresholds compared with NTG (*p* < 0.01 on Days 1–5; *p* < 0.001 on Days 7–9).

### 3.3. PBM Alone or in Combination With SUMA Decreased NTG‐Induced Thermal Hypersensitivity

The results of two‐way repeated‐measures ANOVA of paw withdrawal latency showed significant effects of treatments (*F*_(3.876,  209.3)_ = 85.93, *p* < 0.001), day (*F*_(5,  54)_ = 49.16, *p* < 0.001), and their interaction (*F*_(20,  216)_ = 3.161, *p* < 0.001). The results of the basal response to the thermal stimulus showed no significant difference between the study groups. Intergroup comparisons showed that in the NTG group compared to the sham group, repeated NTG injections increased thermal sensitivity and caused thermal hyperalgesia (Figure 4, *p* < 0.01 on Day 1 and *p* < 0.001 for other test days). However, PBM or SUMA, alone (for both single treatments, *p* < 0.05 on Days 3–7 and *p* < 0.01 on Day 9) or in combination (*p* < 0.05 on Days 1 and 3 and *p* < 0.001 on Days 5 to 9), significantly increased paw withdrawal latency in NTG‐injected mice.

**FIGURE 4:**
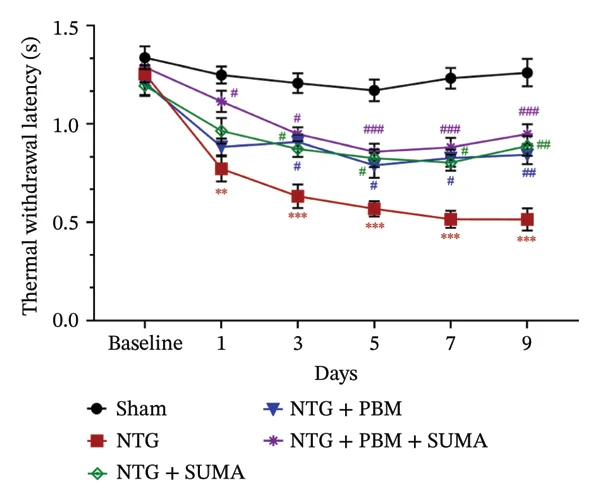
Effects of PBM and SUMA treatments on thermal hypersensitivity in NTG‐induced migraine mouse model. The results are shown as mean ± SEM for 10 animals for each group at different times. ^∗∗^ *p* < 0.01 and ^∗∗∗^ *p* < 0.001 compared to the Sham group. ^#^ *p* < 0.05, ^##^ *p* < 0.01, and ^###^ *p* < 0.001 compared to the NTG group. NTG, nitroglycerin; SUMA, sumatriptan; PBM, photobiomodulation.

### 3.4. PBM Alone or in Combination With SUMA Ameliorated Light‐Aversive Behavior in a Mouse Model of Migraine

In the light/dark box test, significant between‐group differences were observed in initial latency to enter the dark compartment (*F*_(4,  45)_ = 17.31, *p* < 0.001), latency to return the light box (*F*_(4,  45)_ = 19.72, *p* < 0.001), and the light chamber time (*F*_(4,  45)_ = 47.12, *p* < 0.001) across the groups. As shown in Figure 5, repeated NTG injection decreased the latency to enter the dark chamber (*p* < 0.001; Figure 5a), increased the latency to re‐enter the light chamber (*p* < 0.001; Figure 5b), and reduced the percentage of time spent in the light chamber (*p* < 0.001; Figure 5c) compared to the sham mice. The schematic representation of the light/dark box apparatus is shown in Figure 5d. Compared with NTG‐treated mice, PBM, SUMA, and PBM + SUMA treatment increased the latency to enter the dark compartment (*p* < 0.05 for PBM and SUMA; *p* < 0.001 for the combination), increased the percentage of time spent in the light compartment (*p* < 0.001 for all treatments), and reduced the latency to return to the light compartment (*p* < 0.001 for all treatments), consistent with reduced light‐aversive behavior.

**FIGURE 5:**
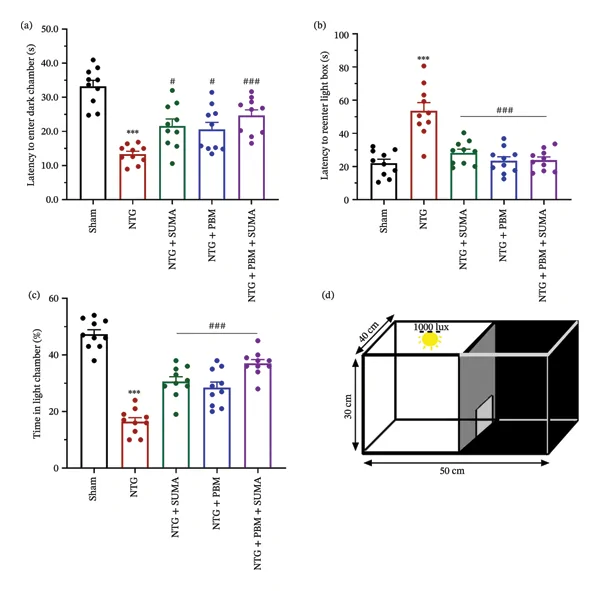
Effects of PBM and SUMA treatment on light‐aversive behavior in NTG‐induced mice. (a) Latency to enter the dark chamber, (b) latency to reenter the light box, (c) the percentage of time spent in the light chamber, and (d) illustration of the light/dark box used for assessment of photophobia. The results are shown as the mean ± SEM for 10 animals per group. ^∗∗∗^ *p* < 0.001 compared to the Sham group. ^#^ *p* < 0.05 and ^###^ *p* < 0.001 compared to the NTG group. NTG, nitroglycerin; SUMA, sumatriptan; PBM, photobiomodulation.

### 3.5. PBM Alone or in Combination With SUMA Downregulated miR155 Expression and Inflammatory Markers in the TNC of NTG‐Induced Mice

To investigate changes in inflammatory signaling in the TNC following repeated NTG administration and treatment, we measured miR‐155‐5p expression and the protein levels of p‐NF‐κB and IL‐1β. One‐way ANOVA revealed significant differences among the experimental groups in miR‐155 expression (*F*_(4,  25)_ = 31.57, *p* < 0.001), p‐NF‐κB (*F*_(4,  10)_ = 22.51, *p* < 0.001), and IL‐1β (*F*_(4,  10)_ = 14.46, *p* < 0.001) protein levels in the TNC. Repeated NTG administration significantly increased miR‐155 expression (*p* < 0.001) and p‐NF‐κB (*p* < 0.01) and IL‐1β (*p* < 0.01) protein levels compared with the sham group (Figure 6a–d). Both PBM and SUMA monotherapies significantly decreased miR‐155 expression compared to the NTG group (*p* < 0.001 and *p* < 0.05, respectively), and the combined PBM + SUMA treatment also produced a significant reduction (*p* < 0.001). Notably, the expression of miR‐155 was significantly lower after the combined treatment than after SUMA (*p* < 0.01) or PBM (*p* < 0.05) monotherapy. For the inflammatory proteins, both PBM and SUMA monotherapies significantly reduced p‐NF‐κB protein levels compared with the NTG group, whereas IL‐1β levels were significantly reduced only following the combined PBM + SUMA treatment. In particular, the levels of p‐NF‐κB (*p* < 0.01) and IL‐1β (*p* < 0.05) proteins diminished compared to the NTG group (Figure 6b–d). Thus, PBM and SUMA were associated with reduced miR‐155 and p‐NF‐κB levels, while the reduction in IL‐1β was observed only with the combined treatment.

**FIGURE 6:**
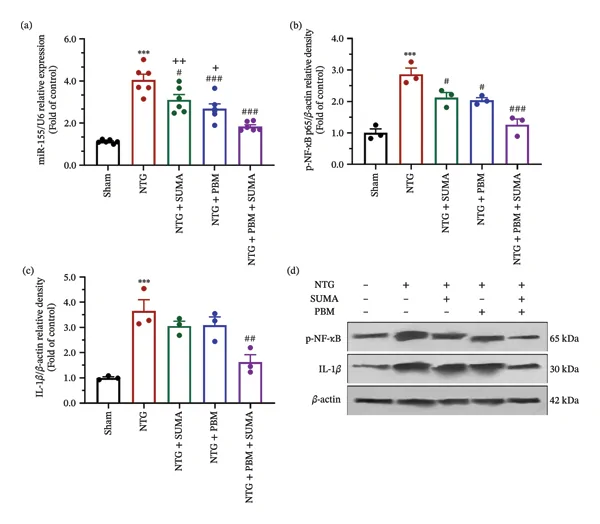
Effects of PBM and SUMA on (a) miR‐155 expression and protein levels of (b) p‐NF‐κB and (c) IL‐1β in the TNC after intermittent NTG injections. (d) Representative Western blot images for each group. The results are shown as mean ± SEM for three or six animals per group. ^∗∗∗^ *p* < 0.001 compared to the Sham group. ^#^ *p* < 0.05, ^##^ *p* < 0.01, and ^###^ *p* < 0.001 compared to the NTG group. ^+^ *p* < 0.05 and ^++^ *p* < 0.01 compared to the NTG + PBM + SUMA. NTG, nitroglycerin; SUMA, sumatriptan; PBM, photobiomodulation; TNC, trigeminal nucleus caudalis.

### 3.6. PBM Alone or Combined With SUMA Decreased Central Sensitization‐Related Proteins in the TNC

Using Western blot analysis, we examined molecular markers associated with trigeminal activation and nociceptive signaling. Specifically, we looked at the expression of key proteins implicated in migraine pathophysiology, comprising c‐Fos (a marker of neuronal activation), TRPA1 and TRPV1 as nociceptive channels, as well as CGRP and PACAP (migraine‐associated neuropeptides).

The results of one‐way ANOVA confirmed remarkable changes in protein levels of c‐Fos (*F*_(4,  10)_ = 68.33, *p* < 0.001), TRPA1 (*F*_(4,  10)_ = 55.71, *p* < 0.001), TRPV1 (*F*_(4,  10)_ = 24.46, *p* < 0.001), CGRP (*F*_(4,  10)_ = 26.86, *p* < 0.001), and PACAP (*F*_(4,  10)_ = 33.51, *p* < 0.001) among the study groups. Further analysis revealed that repeated NTG administration increased c‐Fos, TRPA1, TRPV1, CGRP, and PACAP (all *p* < 0.001; Figure 7a–f) protein levels in TNC. Nevertheless, the combination treatment significantly reduced these protein levels compared with the NTG group (c‐Fos: *p* < 0.01; TRPA1: *p* < 0.001; TRPV1: *p* < 0.001; CGRP: *p* < 0.01; PACAP: *p* < 0.001). Besides, single therapy with PBM or SUMA was effective in reducing TRPA1 (*p* < 0.001 for both monotherapies), TRPV1 (*p* < 0.05 for both monotherapies), CGRP (*p* < 0.01 for SUMA and *p* < 0.05 for PBM), and PACAP (*p* < 0.05 for monotherapy with SUMA) protein expressions compared to the NTG‐received mice.

**FIGURE 7:**
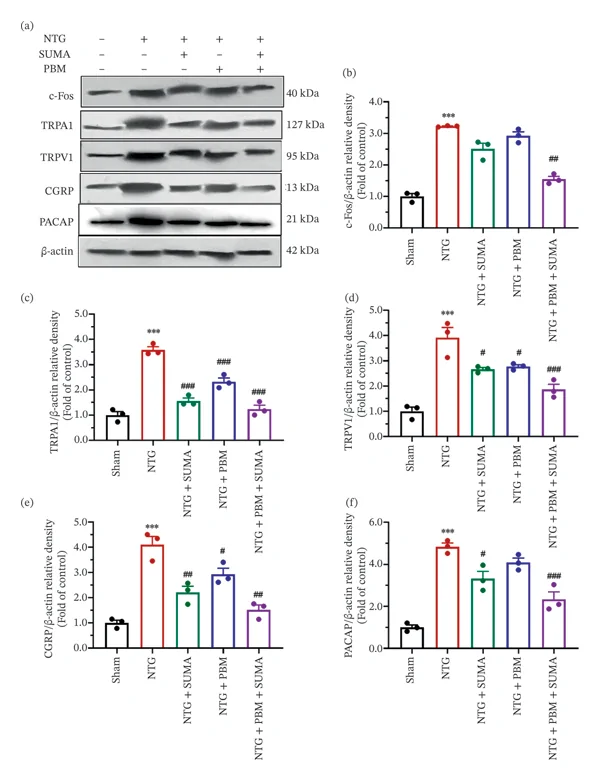
Effects of PBM and SUMA on protein expressions of (b) c‐Fos, (c) TRPA1, (d) TRPV1, (e) CGRP, and (f) PACAP in the TNC of NTG‐induced migraine model. (a) Representative Western blot images for each group. Results are shown as mean ± SEM for three animals per group. ^∗∗∗^ *p* < 0.001 compared to the Sham group. ^#^ *p* < 0.05, ^##^ *p* < 0.01, and ^###^ *p* < 0.001 compared to NTG group. NTG, nitroglycerin; SUMA, sumatriptan; PBM, photobiomodulation; TNC, trigeminal nucleus caudalis; TRPA1, transient receptor potential ankyrin 1; TRPV1, transient receptor potential vanilloid 1; CGRP, calcitonin gene‐related peptide; PACAP, pituitary adenylate cyclase activating polypeptide.

## 4. Discussion

This study provides the first preclinical evidence that transcranial PBM, alone or in combination with SUMA, reduced mechanical and thermal hypersensitivity, photophobia, and spontaneous pain‐related behaviors in an NTG‐induced mouse model of trigeminovascular system‐induced migraine. Moreover, PBM lowered TNC miR‐155 expression, inflammatory mediator levels, and migraine‐related neuropeptides. Figure 8 provides a schematic overview of the NTG‐induced mouse model and the behavioral and molecular changes associated with PBM treatment.

**FIGURE 8:**
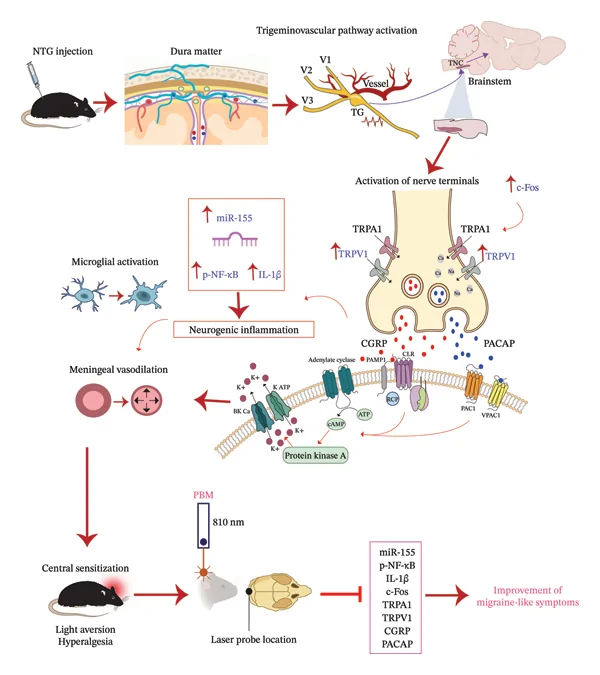
Schematic illustration of the NTG‐induced mouse model of migraine and the biochemical mediators involved in the pathogenesis. PBM delivered by an 808 nm laser to the head improves the migraine‐like symptoms and reverses the biochemical changes, particularly when combined with SUMA. NTG, nitroglycerin; TG, trigeminal ganglion; TNC, trigeminal nucleus caudalis; PBM, photobiomodulation; SUMA, sumatriptan; TRPA1, transient receptor potential ankyrin 1; TRPV1, transient receptor potential vanilloid 1; CGRP, calcitonin gene‐related peptide; and PACAP, pituitary adenylate cyclase activating polypeptide.

Repeated systemic NTG administration activates the trigeminovascular system and produces a range of migraine‐like behavioral and molecular changes in rodents, including trigeminal ganglion and TNC hypersensitivity, reduced locomotor activity, photophobia‐like behavior, and heightened thermal and mechanical sensitivity [14, 15, 42–45]. However, the NTG model reproduces selected features of migraine and does not fully capture the clinical heterogeneity of episodic and chronic migraine. Mechanistically, NTG causes meningeal vasodilation and activates TRPV1 channels, both directly, through nitrosylation of cysteine residues on the channel proteins, and indirectly, through the release of inflammatory mediators that promote sensory nerve depolarization. This initial activation of peripheral trigeminal afferents is further amplified by TRPV1‐/TRPA1‐mediated release of CGRP and PACAP from sensory nerve endings, thereby promoting vasodilation, vascular permeability, and neurogenic inflammation in a self‐perpetuating cycle [7–11, 42]. Importantly, the resulting inflammatory signaling can extend to the central trigeminal system, where neurogenic inflammation and glial activation in the TNC contribute to NTG‐induced hyperalgesia by lowering activation thresholds and increasing trigeminal pathway excitability [17, 46–50].

Consistent with previous studies [14, 15, 44, 48], we found that five NTG injections given every other day gradually reduced paw and periorbital mechanical sensitivity thresholds and paw thermal withdrawal latency before and 2 hours after the injection, indicating the development of persistent basal hypersensitivity accompanied by acute hyperalgesia. In parallel with these nociceptive responses, repeated NTG administration produced light aversion, reflected by a decreased latency to enter the dark chamber and an increased latency to re‐enter the light compartment, consistent with photophobia‐like behavior reported in previous experimental migraine models [14, 15, 43, 44]. These behavioral findings support the reproducibility of the NTG‐induced migraine‐like phenotype. Moreover, chronic NTG‐treated mice exhibited reduced locomotor and exploratory activity together with increased grooming and head scratching. Consistent with our findings, repeated NTG administration has been shown to reduce locomotor and exploratory activity while increasing grooming and other spontaneous pain‐related behaviors in rodents, supporting their value as complementary measures of the behavioral phenotype associated with experimental migraine [44, 51, 52]. These spontaneous behavioral responses are not migraine‐specific and may also be influenced by general activity or stress. However, they should be interpreted in conjunction with more migraine‐relevant outcomes, such as cephalic hypersensitivity and photophobia‐like behavior, and should be regarded as complementary measures rather than as standalone indicators of migraine‐like behavior.

On the other hand, PBM and SUMA treatments, particularly when administered together, were associated with greater improvements in several behavioral outcomes than either intervention alone. These findings are consistent with previous evidence supporting the analgesic effects of PBM in several pain conditions, including rheumatoid arthritis, neuropathic pain, musculoskeletal pain, fibromyalgia, and orofacial pain [25]. Although evidence regarding PBM in migraine remains limited, previous clinical studies have reported the beneficial effects of laser therapy or PBM on migraine‐related outcomes. Allais et al. reported the benefits of laser therapy at 904 nm in reducing headache days in chronic migraine sufferers [53]. In a clinical trial, PBM at 808 nm (twice a week, 10 sessions) was found to be as efficient as botulinum toxin A in lessening medication consumption, migraine days, and pain severity [54]. Although the combination treatment resulted in greater effects than individual treatments for several outcomes, the present experimental design and analyses do not determine whether these effects were additive, synergistic, or independent. Therefore, the combined results are interpreted as higher effectiveness relative to the individual interventions, rather than as evidence of pharmacological synergism. Nevertheless, these results provide a rationale for additional research into the potential of PBM as an adjunctive approach to conventional pharmacotherapy.

The behavioral effects of NTG and their attenuation by PBM were accompanied by changes in inflammatory signaling within the TNC. The noncoding RNA miR‐155 serves as a crucial regulator of neuroinflammation by activating NF‐κB signaling, inducing pro‐inflammatory cytokines, and inhibiting anti‐inflammatory mediators [55]. Clinical studies reported elevated blood levels of miR‐155 [56, 57], TNF‐*α*, and IL‐1*β* [58, 59] in episodic and chronic migraineurs. In addition, NTG‐induced chronic migraine models show elevated miR‐155 in serum and the trigeminal system, along with hyperalgesia and increased cytokines and neuropeptides such as CGRP, all of which are reversed by miR‐155 or CGRP antagonists [17, 60]. Tan et al. showed that miR‐155 suppression reduced thermal and mechanical hypersensitivity and NF‐κB levels in a neuropathic pain model [61]. Recently, Sun et al. showed that the inhibition of NF‐κB reduces c‐Fos and CGRP in the TNC and increases mechanical pain thresholds in a recurring migraine model [46]. In line with this evidence, our study showed that NTG increased miR‐155, p‐NF‐κB, and IL‐1*β* levels in the TNC and induced hindpaw and facial hyperalgesia, indicating an association between altered TNC inflammatory signaling and the NTG‐induced migraine‐like phenotype.

Similar to SUMA, PBM reduced miR‐155 and p‐NF‐κB levels in the TNC of NTG‐treated mice. These findings are consistent with the reported anti‐inflammatory effects of PBM, which include reductions in pro‐inflammatory cytokines, inhibition of prostaglandin E2 and cyclooxygenase‐2, and enhancement of anti‐inflammatory cytokines [62]. In addition, PBM efficiently inhibits p38 MAPK phosphorylation [63], which plays a crucial role in activating the NF‐κB signaling pathway [64]. The observed reduction in miR‐155 is of particular interest given the reported involvement of miR‐155 in pro‐inflammatory signaling and microglial responses following NTG administration [17, 60]. Previous studies have also suggested that PBM can influence inflammatory and nociceptive pathways. For instance, Oliveira et al. demonstrated that PBM attenuated nociceptive responses in association with reduced inflammatory mediators and decreased glial activation‐related changes in the dorsal root ganglia [65]. Similarly, 660 nm PBM reduced fractalkine (CX3CL1) expression and orofacial nociceptive responses in a temporomandibular pain model [66]. Together, these findings support the possibility that modulation of inflammatory signaling, including the miR‐155/NF‐κB axis, contributes to the behavioral effects of PBM, although the present findings demonstrate an association rather than a causal relationship. Since neither miR‐155 nor NF‐κB signaling was directly manipulated in this study, the molecular pathway mediating behavioral effects of PBM remains to be established.

In addition to inflammatory signaling, our findings indicate that repeated NTG administration enhanced trigeminal neuronal activation and neuropeptide‐associated signaling, whereas PBM attenuated these molecular changes. Increased synaptic transmission and central sensitization in migraine are linked to c‐Fos upregulation, a hallmark of nociceptive pathway activation [67, 68]. In line with earlier studies [17, 46, 69, 70], NTG injection elevated c‐Fos and central sensitization‐related proteins, including TRPA1 and TRPV1, as well as the migraine‐related neuropeptides CGRP and PACAP, in the TNC, indicating enhanced trigeminal activation and neuropeptide signaling. These molecular changes are consistent with activation of trigeminal nociceptive pathways, in which TRPV1‐/TRPA1‐positive afferents can facilitate the release of CGRP and PACAP, thereby contributing to neurogenic inflammation, vascular responses, and nociceptive sensitization. Therefore, the elevated levels of these proteins after repeated NTG administration might be indicative of an association between trigeminal activation and the behavioral hypersensitivity in this model rather than a direct causal mechanism.

Nevertheless, we found that PBM reduced c‐Fos, TRPV1, CGRP, and PACAP protein expression in the TNC. Consistent with our findings, chronic 904 nm PBM reduced CGRP, substance P, and TRPV1 in the trigeminal ganglia of a temporomandibular pain model [71]. These findings are consistent with the proposed multifaceted analgesic actions of PBM, which include modulation of TRPV1 channels, inhibition of substance P signaling, and other effects on nociceptive pathways [72, 73]. In this regard, Ryu et al. reported that laser therapy exerted analgesic effects through inhibition of TRPV1 channels [74]. Another study showed that PBM at 904 nm downregulated substance P and TRPV1 proteins in the dorsal root ganglia in a sciatic nerve injury model [32]. Zupin et al. also found that PBM could reduce capsaicin‐induced pain by inhibition of TRPV1 channel activity and modulation of mitochondrial metabolism [25]. In a rat temporomandibular pain model, 20 days of 904 nm PBM increased allodynia thresholds by downregulating CGRP, substance P, and TRPV1 in the trigeminal ganglion [71]. Taken together, these results suggest that attenuation of trigeminal activation and neuropeptide signaling induced by PBM may be involved in the reduction of pain‐related responses in the NTG model.

There are currently no established or optimized irradiation parameters for carrying out PBM in rodent migraine models. In this study, we selected 810 nm based on prior research showing that its transcranial application significantly increased pain thresholds, with effects peaking at 2–3 h and lasting up to 24 h [36, 37]. The 810 nm wavelength is widely used in transcranial PBM for its strong cranial penetration and optimal absorption by cytochrome c oxidase (CCO) [24, 75]. Reinhart et al. found that 810‐nm light reached approximately 5 mm deep in the brains of male BALB/*c* mice, sufficient to reach the substantia nigra pars compacta [76]. Moreover, several studies have demonstrated the efficacy of 810‐nm PBM in reducing pain and inflammation [77], further supporting the rationale for selecting this wavelength in the present migraine model. In addition to wavelength, the fluence and temporal characteristics of light delivery are important determinants of PBM responses. Previous transcranial PBM studies in rodents have suggested that fluences ≤ 10 J/cm^2^ are generally within a nonthermal range, whereas higher fluences (> 20 J/cm^2^) may increase the risk of tissue damage, while lower fluences (< 5 J/cm^2^) may be less effective [24]. Consequently, the fluence of 8 J/cm^2^ was chosen in accordance with previously reported transcranial PBM studies in rodents and is within the experimental ranges that are frequently employed to investigate neuromodulatory effects [34, 78]. Nevertheless, the safety of this particular irradiation protocol cannot be determined from our findings due to the absence of a systematic assessment of tissue temperature and histological indicators of tissue injury in the present study. The present study employs 10‐Hz pulsed irradiation, which intermittently delivers energy, potentially reducing continuous energy deposition and tissue heating [38, 77]. Although the effective penetration depth may be improved by increasing the power, the risk of thermal damage is also increased [24]. Together, these parameters were chosen based on previously reported experimental PBM protocols and should be regarded as exploratory rather than optimized. Additional research is required to establish safe and effective irradiation parameters for transcranial PBM in migraine models.

There are several limitations of this study that need to be acknowledged. First, the study was done in male mice only, so the results cannot be generalized to both sexes, particularly since migraine is more common in females. To ascertain whether the therapeutic effects of PBM differ between males and females, future studies should involve both sexes and evaluate sex‐by‐treatment interactions. Second, although repeated NTG administration replicates a number of migraine characteristics, including trigeminal hypersensitivity, photophobia, and altered inflammatory signaling, the model does not fully capture the clinical heterogeneity of episodic and chronic migraine. Further validation in complementary preclinical models is warranted to evaluate the generalizability of these findings beyond the NTG model. Third, molecular analyses were restricted to the TNC and were not performed in other migraine‐relevant regions, such as trigeminal ganglia, meninges, hypothalamus, or cortex. Therefore, the impact of PBM on inflammatory and trigeminal signaling in the broader migraine‐related network is still uncertain. Future research that incorporates molecular and functional evaluations of these regions may offer a deeper understanding of the mechanisms and effects of PBM. In addition, photophobia was only assessed at the end of the experiment. Although this method may minimize the risk of habituation to the light/dark apparatus, long‐term assessments that employ alternative paradigms would offer a more comprehensive understanding of the acute and cumulative effects of PBM. Furthermore, the current design is unable to investigate whether PBM at the selected parameters alters basal nociceptive or behavioral responses in the absence of NTG‐induced migraine‐like pathophysiology, as a healthy PBM‐treated control group was not included. Subsequent investigations that involve healthy PBM‐treated animals will be crucial in determining the physiological significance and specificity of the observed PBM effects. Lastly, the Western blot analyses were performed with a relatively small sample size (*n* = 3/group). These molecular findings will need to be validated in larger cohorts to establish statistical robustness and reproducibility.

## 5. Conclusion

This study presents preclinical evidence indicating that transcranial PBM diminishes pain‐related behavioral responses and lowers neuroinflammatory mediators and migraine‐related neuropeptides in the TNC of NTG‐treated mice. The beneficial effects of PBM were observed both alone and in combination with SUMA, with the combination showing greater efficacy than either treatment alone for several behavioral and molecular outcomes. These findings support further investigation of transcranial PBM as a potential adjunctive strategy for migraine management.

## Author Contributions

S.K. and S.M.V.: conceptualization, methodology, data curation, and behavioral experiments; S.Z.A., A.E., and A.M.: conceptualization, methodology, validation, and writing–review and editing; J.M., S.S.‐E., and M.R.: conceptualization, methodology, supervision, formal analysis, and writing–review and editing; S.A.‐S. R.N.‐A., A.N., M.H., and L.H.: investigation, behavioral experiments, and data curation; M.R.H.: methodology, photobiomodulation expertise, laser parameter setup, and supervision; F.F. conceptualization, funding acquisition, project administration, supervision, data analysis, validation, writing–original draft, and writing–review and editing.

## Funding

This study was supported by grants from Tabriz University of Medical Sciences, Tabriz, Iran (Grants 67229, 66770, and 73151).

## Disclosure

All authors critically reviewed and approved the final version of the manuscript.

## Conflicts of Interest

The authors declare no conflicts of interest.

## Supporting Information

Additional supporting information can be found online in the Supporting Information section.

## Supporting information


**Supporting Information** Supporting Figure S1: Original, uncropped Western blot images corresponding to Figures 6 and 7. Supporting Tables S1–S3: Detailed statistical analyses, including effect‐size estimates and 95% confidence intervals for pairwise mean differences.

## Data Availability

The data that support the findings of this study are available within the article and its supporting Information.
