# Supplementary material for: Transcranial Photobiomodulation Attenuates Neurogenic Inflammation and Sensory Hypersensitivity in a Nitroglycerin‐Induced Migraine Model: Toward a Nonpharmacological Therapy
Source: Pain Res Manag. 2026 Oct 17;2026:1530362. doi: 10.1155/prm/1530362 (PMC13675831; doi:10.1155/prm/1530362)
Supplement: Supplementary file 1 [file PRM-2026-1530362-s001.zip › Supplementary Tables S1-S3.docx]

**Supplementary Table S1. Detailed statistical analysis of repeated-measures behavioral outcomes**

**Part A. Omnibus effects and effect-size estimates from repeated-measures ANOVA of behavioral outcomes**

| **Outcome** | **Effect** | **Greenhouse–Geisser ε** | **Partial η²** |
| --- | --- | --- | --- |
| Hindpaw mechanical threshold | Treatment | 1.000 | 0.8462 |
| Hindpaw mechanical threshold | Time | 0.817 | 0.0817 |
| Hindpaw mechanical threshold | Time × Treatment | 0.817 | 0.0283 |
| Hindpaw basal mechanical threshold | Treatment | 1.000 | 0.8671 |
| Hindpaw basal mechanical threshold | Time | 0.763 | 0.3291 |
| Hindpaw basal mechanical threshold | Time × Treatment | 0.763 | 0.2019 |
| Periorbital mechanical threshold | Treatment | 1.000 | 0.8227 |
| Periorbital mechanical threshold | Time | 0.852 | 0.02586 |
| Periorbital mechanical threshold | Time × Treatment | 0.852 | 0.01827 |
| Periorbital basal mechanical threshold | Treatment | 1.000 | 0.7395 |
| Periorbital basal mechanical threshold | Time | 0.804 | 0.243 |
| Periorbital basal mechanical threshold | Time × Treatment | 0.804 | 0.1767 |
| Thermal nociception | Treatment | 1.000 | 0.8832 |
| Thermal nociception | Time | 0.831 | 0.5136 |
| Thermal nociception | Time × Treatment | 0.831 | 0.2216 |
| Head scratching | Treatment | 1.000 | 0.724 |
| Head scratching | Time | 0.866 | 0.4282 |
| Head scratching | Time × Treatment | 0.866 | 0.4449 |
| Distance traveled | Treatment | 1.000 | 0.8017 |
| Distance traveled | Time | 0.792 | 0.7601 |
| Distance traveled | Time × Treatment | 0.792 | 0.4686 |
| Grooming time | Treatment | 1.000 | 0.7399 |
| Grooming time | Time | 0.906 | 0.3475 |
| Grooming time | Time × Treatment | 0.906 | 0.5039 |
| Exploratory activity | Treatment | 1.000 | 0.7149 |
| Exploratory activity | Time | 0.784 | 0.6901 |
| Exploratory activity | Time × Treatment | 0.784 | 0.5874 |
|  |  |  |  |

**Part B. Pairwise comparisons, adjusted P values, mean differences, and 95% CIs**

| **Outcome** | **Comparison** | **Mean difference (Group 2 − Group 1)** | **95% CI** | **Adjusted P value** |
| --- | --- | --- | --- | --- |
| Hindpaw mechanical threshold | Sham vs NTG | <0.0005 | -1.665 to -0.819 | 0.001 |
| Hindpaw mechanical threshold | NTG vs NTG+SUMA | 0.462 | 0.039 to 0.885 | 0.0245 |
| Hindpaw mechanical threshold | NTG vs NTG+PBM | 0.442 | 0.019 to 0.865 | 0.03556 |
| Hindpaw mechanical threshold | NTG vs NTG+PBM+SUMA | 0.542 | 0.119 to 0.965 | 0.004696 |
| Hindpaw mechanical threshold | NTG+SUMA vs NTG+PBM+SUMA | 0.08 | -0.343 to 0.503 | 0.9 |
| Hindpaw mechanical threshold | NTG+PBM vs NTG+PBM+SUMA | 0.1 | -0.323 to 0.523 | 0.9 |
| Hindpaw basal mechanical threshold | Sham vs NTG | <0.0005 | -1.996 to -0.954 | 0.001 |
| Hindpaw basal mechanical threshold | NTG vs NTG+SUMA | 0.575 | 0.054 to 1.096 | 0.02249 |
| Hindpaw basal mechanical threshold | NTG vs NTG+PBM | 0.471 | -0.050 to 0.992 | 0.09723 |
| Hindpaw basal mechanical threshold | NTG vs NTG+PBM+SUMA | 0.835 | 0.314 to 1.356 | 0.001 |
| Hindpaw basal mechanical threshold | NTG+SUMA vs NTG+PBM+SUMA | 0.26 | -0.261 to 0.781 | 0.626 |
| Hindpaw basal mechanical threshold | NTG+PBM vs NTG+PBM+SUMA | 0.364 | -0.157 to 0.885 | 0.308 |
| Periorbital mechanical threshold | Sham vs NTG | <0.0005 | -0.844 to -0.370 | 0.001 |
| Periorbital mechanical threshold | NTG vs NTG+SUMA | 0.279 | 0.042 to 0.516 | 0.01216 |
| Periorbital mechanical threshold | NTG vs NTG+PBM | 0.283 | 0.046 to 0.520 | 0.0105 |
| Periorbital mechanical threshold | NTG vs NTG+PBM+SUMA | 0.423 | 0.186 to 0.660 | 0.001 |
| Periorbital mechanical threshold | NTG+SUMA vs NTG+PBM+SUMA | 0.144 | -0.093 to 0.381 | 0.4544 |
| Periorbital mechanical threshold | NTG+PBM vs NTG+PBM+SUMA | 0.14 | -0.097 to 0.377 | 0.4827 |
| Periorbital basal mechanical threshold | Sham vs NTG | <0.0005 | -0.964 to -0.392 | 0.001 |
| Periorbital basal mechanical threshold | NTG vs NTG+SUMA | 0.35 | 0.064 to 0.636 | 0.007908 |
| Periorbital basal mechanical threshold | NTG vs NTG+PBM | 0.306 | 0.020 to 0.592 | 0.02908 |
| Periorbital basal mechanical threshold | NTG vs NTG+PBM+SUMA | 0.374 | 0.088 to 0.660 | 0.003625 |
| Periorbital basal mechanical threshold | NTG+SUMA vs NTG+PBM+SUMA | 0.024 | -0.262 to 0.310 | 0.9 |
| Periorbital basal mechanical threshold | NTG+PBM vs NTG+PBM+SUMA | 0.068 | -0.218 to 0.354 | 0.9 |
| Head scratching | Sham vs NTG | 3.75 | 2.441 to 5.059 | 0.001 |
| Head scratching | NTG vs NTG+SUMA | <0.0005 | -4.934 to -2.316 | 0.001 |
| Head scratching | NTG vs NTG+PBM | <0.0005 | -4.684 to -2.066 | 0.001 |
| Head scratching | NTG vs NTG+PBM+SUMA | <0.0005 | -6.184 to -3.566 | 0.001 |
| Head scratching | NTG+SUMA vs NTG+PBM+SUMA | <0.0005 | -2.559 to 0.059 | 0.06828 |
| Head scratching | NTG+PBM vs NTG+PBM+SUMA | <0.0005 | -2.809 to -0.191 | 0.01665 |
| Distance traveled | Sham vs NTG | <0.0005 | -1469.125 to -696.500 | 0.001 |
| Distance traveled | NTG vs NTG+SUMA | 257.6 | -128.725 to 643.900 | 0.3448 |
| Distance traveled | NTG vs NTG+PBM | 272.1 | -114.212 to 658.412 | 0.2903 |
| Distance traveled | NTG vs NTG+PBM+SUMA | 450.7 | 64.400 to 837.025 | 0.01411 |
| Distance traveled | NTG+SUMA vs NTG+PBM+SUMA | 193.1 | -193.187 to 579.437 | 0.6136 |
| Distance traveled | NTG+PBM vs NTG+PBM+SUMA | 178.6 | -207.700 to 564.925 | 0.6725 |
| Grooming time | Sham vs NTG | 29.02 | 18.940 to 39.110 | 0.001 |
| Grooming time | NTG vs NTG+SUMA | <0.0005 | -29.010 to -8.840 | 0.001 |
| Grooming time | NTG vs NTG+PBM | <0.0005 | -25.797 to -5.628 | 0.001 |
| Grooming time | NTG vs NTG+PBM+SUMA | <0.0005 | -30.097 to -9.928 | 0.001 |
| Grooming time | NTG+SUMA vs NTG+PBM+SUMA | <0.0005 | -11.172 to 8.997 | 0.9 |
| Grooming time | NTG+PBM vs NTG+PBM+SUMA | <0.0005 | -14.385 to 5.785 | 0.7288 |
| Exploratory activity | Sham vs NTG | <0.0005 | -93.007 to -50.443 | 0.001 |
| Exploratory activity | NTG vs NTG+SUMA | 26.18 | 4.893 to 47.457 | 0.008365 |
| Exploratory activity | NTG vs NTG+PBM | 25.09 | 3.806 to 46.369 | 0.01279 |
| Exploratory activity | NTG vs NTG+PBM+SUMA | 35.61 | 14.331 to 56.894 | 0.001 |
| Exploratory activity | NTG+SUMA vs NTG+PBM+SUMA | 9.438 | -11.844 to 30.719 | 0.702 |
| Exploratory activity | NTG+PBM vs NTG+PBM+SUMA | 10.52 | -10.757 to 31.807 | 0.622 |

Part A presents omnibus ANOVA effects and corresponding effect-size estimates. Partial eta squared (ηp²) is reported as an effect-size measure for omnibus effects. For repeated-measures outcomes, Greenhouse–Geisser ε is reported for within-subject effects when applicable. Part B presents Tukey-adjusted pairwise comparisons, including mean differences and 95% confidence intervals. F statistics, degrees of freedom, and omnibus P values are reported in the main manuscript.

**Supplementary Table S2. Statistical details for single-time-point behavioral outcomes**

**Part A. Omnibus effects and effect sizes for other behavioral outcomes**

| **Outcome** | **Partial η²** |
| --- | --- |
| Light first entrance | 0.6061 |
| Light duration | 0.8073 |
| Latency to enter light box | 0.6368 |

**Part B. Pairwise comparisons for other behavioral outcomes**

| **Outcome** | **Comparison** | **Mean difference (Group 2 − Group 1)** | **95% CI** | **Adjusted P value** |
| --- | --- | --- | --- | --- |
| Light first entrance | Sham vs NTG | <0.0005 | -26.894 to -12.948 | 0.001 |
| Light first entrance | NTG vs NTG+SUMA | 8.28 | 1.307 to 15.253 | 0.01266 |
| Light first entrance | NTG vs NTG+PBM | 7.276 | 0.303 to 14.249 | 0.03698 |
| Light first entrance | NTG vs NTG+PBM+SUMA | 11.32 | 4.346 to 18.292 | 0.001 |
| Light first entrance | NTG+SUMA vs NTG+PBM+SUMA | 3.039 | -3.934 to 10.012 | 0.7048 |
| Light first entrance | NTG+PBM vs NTG+PBM+SUMA | 4.043 | -2.930 to 11.016 | 0.4768 |
| Light duration | Sham vs NTG | <0.0005 | -37.558 to -24.242 | 0.001 |
| Light duration | NTG vs NTG+SUMA | 14.2 | 7.542 to 20.858 | 0.001 |
| Light duration | NTG vs NTG+PBM | 12 | 5.342 to 18.658 | 0.001 |
| Light duration | NTG vs NTG+PBM+SUMA | 20.6 | 13.942 to 27.258 | 0.001 |
| Light duration | NTG+SUMA vs NTG+PBM+SUMA | 6.4 | -0.258 to 13.058 | 0.06497 |
| Light duration | NTG+PBM vs NTG+PBM+SUMA | 8.6 | 1.942 to 15.258 | 0.005504 |
| Latency to enter light box | Sham vs NTG | 31.71 | 19.674 to 43.754 | 0.001 |
| Latency to enter light box | NTG vs NTG+SUMA | <0.0005 | -37.511 to -13.431 | 0.001 |
| Latency to enter light box | NTG vs NTG+PBM | <0.0005 | -42.239 to -18.159 | 0.001 |
| Latency to enter light box | NTG vs NTG+PBM+SUMA | <0.0005 | -41.841 to -17.761 | 0.001 |
| Latency to enter light box | NTG+SUMA vs NTG+PBM+SUMA | <0.0005 | -16.370 to 7.710 | 0.8248 |
| Latency to enter light box | NTG+PBM vs NTG+PBM+SUMA | 0.398 | -11.642 to 12.438 | 0.9 |

Part A presents omnibus ANOVA effects and corresponding effect-size estimates. Partial eta squared (ηp²) is reported as an effect-size measure for omnibus effects. Part B presents Tukey-adjusted pairwise comparisons, including mean differences and 95% confidence intervals. F statistics, degrees of freedom, and omnibus P values are reported in the main manuscript.

**Supplementary Table S3. Detailed statistical analysis of molecular outcomes in the trigeminal nucleus caudalis**

**Part A. Omnibus effects and effect sizes for molecular outcomes**

| **Outcome** | **Partial η²** |
| --- | --- |
| c-Fos | 0.8325 |
| CGRP | 0.792 |
| PACAP | 0.8482 |
| TRPV1 | 0.9133 |
| IL-1β | 0.7834 |
| NF-κB | 0.7906 |
| TRPA1 | 0.9571 |
| miR-155 | 0.8348 |
|  |  |

**Part B. Pairwise comparisons for molecular outcomes**

| **Outcome** | **Comparison** | **Mean difference (Group 2 − Group 1)** | **95% CI** | **Adjusted P value** |
| --- | --- | --- | --- | --- |
| c-Fos | Sham vs NTG | 1.535 | 0.749 to 2.321 | 0.001 |
| c-Fos | NTG vs NTG+SUMA | <0.0005 | -1.430 to 0.142 | 0.1243 |
| c-Fos | NTG vs NTG+PBM | <0.0005 | -1.453 to 0.119 | 0.1078 |
| c-Fos | NTG vs NTG+PBM+SUMA | <0.0005 | -2.028 to -0.456 | 0.002854 |
| c-Fos | NTG+SUMA vs NTG+PBM+SUMA | <0.0005 | -1.384 to 0.188 | 0.1656 |
| c-Fos | NTG+PBM vs NTG+PBM+SUMA | <0.0005 | -1.361 to 0.211 | 0.1896 |
| CGRP | Sham vs NTG | 1.792 | 0.774 to 2.810 | 0.001273 |
| CGRP | NTG vs NTG+SUMA | <0.0005 | -2.089 to -0.054 | 0.03817 |
| CGRP | NTG vs NTG+PBM | <0.0005 | -2.172 to -0.136 | 0.02524 |
| CGRP | NTG vs NTG+PBM+SUMA | <0.0005 | -2.476 to -0.440 | 0.005734 |
| CGRP | NTG+SUMA vs NTG+PBM+SUMA | <0.0005 | -1.404 to 0.631 | 0.7071 |
| CGRP | NTG+PBM vs NTG+PBM+SUMA | <0.0005 | -1.322 to 0.714 | 0.8443 |
| PACAP | Sham vs NTG | 1.87 | 1.001 to 2.738 | 0.001 |
| PACAP | NTG vs NTG+SUMA | <0.0005 | -1.561 to 0.177 | 0.1389 |
| PACAP | NTG vs NTG+PBM | <0.0005 | -1.703 to 0.034 | 0.06118 |
| PACAP | NTG vs NTG+PBM+SUMA | <0.0005 | -2.162 to -0.425 | 0.004377 |
| PACAP | NTG+SUMA vs NTG+PBM+SUMA | <0.0005 | -1.470 to 0.267 | 0.2284 |
| PACAP | NTG+PBM vs NTG+PBM+SUMA | <0.0005 | -1.328 to 0.410 | 0.4556 |
| TRPV1 | Sham vs NTG | 2.203 | 1.456 to 2.951 | 0.001 |
| TRPV1 | NTG vs NTG+SUMA | <0.0005 | -1.527 to -0.032 | 0.0402 |
| TRPV1 | NTG vs NTG+PBM | <0.0005 | -1.617 to -0.121 | 0.02187 |
| TRPV1 | NTG vs NTG+PBM+SUMA | <0.0005 | -2.222 to -0.726 | 0.001 |
| TRPV1 | NTG+SUMA vs NTG+PBM+SUMA | <0.0005 | -1.442 to 0.053 | 0.07198 |
| TRPV1 | NTG+PBM vs NTG+PBM+SUMA | <0.0005 | -1.353 to 0.143 | 0.1307 |
| IL-1β | Sham vs NTG | 2.193 | 0.788 to 3.598 | 0.003134 |
| IL-1β | NTG vs NTG+SUMA | <0.0005 | -1.816 to 0.994 | 0.8546 |
| IL-1β | NTG vs NTG+PBM | <0.0005 | -2.082 to 0.728 | 0.5344 |
| IL-1β | NTG vs NTG+PBM+SUMA | <0.0005 | -3.056 to -0.245 | 0.02053 |
| IL-1β | NTG+SUMA vs NTG+PBM+SUMA | <0.0005 | -2.645 to 0.166 | 0.09092 |
| IL-1β | NTG+PBM vs NTG+PBM+SUMA | <0.0005 | -2.379 to 0.432 | 0.2278 |
| NF-κB | Sham vs NTG | 1.748 | 0.732 to 2.765 | 0.001517 |
| NF-κB | NTG vs NTG+SUMA | <0.0005 | -2.141 to -0.109 | 0.02896 |
| NF-κB | NTG vs NTG+PBM | <0.0005 | -2.267 to -0.234 | 0.01547 |
| NF-κB | NTG vs NTG+PBM+SUMA | <0.0005 | -2.501 to -0.469 | 0.004993 |
| NF-κB | NTG+SUMA vs NTG+PBM+SUMA | <0.0005 | -1.376 to 0.656 | 0.7496 |
| NF-κB | NTG+PBM vs NTG+PBM+SUMA | <0.0005 | -1.251 to 0.782 | 0.9 |
| TRPA1 | Sham vs NTG | 2.587 | 1.934 to 3.240 | 0.001 |
| TRPA1 | NTG vs NTG+SUMA | <0.0005 | -2.679 to -1.373 | 0.001 |
| TRPA1 | NTG vs NTG+PBM | <0.0005 | -1.916 to -0.610 | 0.001 |
| TRPA1 | NTG vs NTG+PBM+SUMA | <0.0005 | -3.003 to -1.697 | 0.001 |
| TRPA1 | NTG+SUMA vs NTG+PBM+SUMA | <0.0005 | -0.977 to 0.329 | 0.5101 |
| TRPA1 | NTG+PBM vs NTG+PBM+SUMA | <0.0005 | -1.740 to -0.434 | 0.00194 |
| miR-155 | Sham vs NTG | 2.926 | 2.090 to 3.762 | 0.001 |
| miR-155 | NTG vs NTG+SUMA | <0.0005 | -1.784 to -0.112 | 0.02062 |
| miR-155 | NTG vs NTG+PBM | <0.0005 | -2.207 to -0.535 | 0.001 |
| miR-155 | NTG vs NTG+PBM+SUMA | <0.0005 | -3.048 to -1.377 | 0.001 |
| miR-155 | NTG+SUMA vs NTG+PBM+SUMA | <0.0005 | -2.100 to -0.429 | 0.001362 |
| miR-155 | NTG+PBM vs NTG+PBM+SUMA | <0.0005 | -1.677 to -0.006 | 0.04789 |

Part A presents omnibus one-way ANOVA effects and corresponding effect-size estimates. Partial eta squared (ηp²) is reported as an effect-size measure for omnibus effects. Part B presents Tukey-adjusted pairwise comparisons, including mean differences and 95% confidence intervals. F statistics, degrees of freedom, and omnibus P values are reported in the main manuscript.
